# Structure of the multiple functional domains from coronavirus nonstructural protein 3

**DOI:** 10.1080/22221751.2020.1865840

**Published:** 2021-01-17

**Authors:** Mengxia Li, Gang Ye, Yu Si, Zhou Shen, Zhu Liu, Yuejun Shi, Shaobo Xiao, Zhen F. Fu, Guiqing Peng

**Affiliations:** a State Key Laboratory of Agricultural Microbiology, College of Veterinary Medicine, Huazhong Agricultural University, Wuhan, People’s Republic of China; b Key Laboratory of Preventive Veterinary Medicine in Hubei Province, The Cooperative Innovation Center for Sustainable Pig Production, Wuhan, People’s Republic of China; c National Key Laboratory of Crop Genetic Improvement, College of Life Science and Technology, Huazhong Agricultural University, Wuhan, People’s Republic of China

**Keywords:** Multiple functional domains, macro domain, ubiquitin-like domain, papain-like protein, enzyme activity

## Abstract

Coronaviruses (CoVs) are potential pandemic pathogens that can infect a variety of hosts and cause respiratory, enteric, hepatic and neurological diseases. Nonstructural protein 3 (nsp3), an essential component of the replication/transcription complex, is one of the most important antiviral targets. Here, we report the first crystal structure of multiple functional domains from porcine delta-coronavirus (PDCoV) nsp3, including the macro domain (Macro), ubiquitin-like domain 2 (Ubl2) and papain-like protease (PLpro) catalytic domain. In the asymmetric unit, two of the subunits form the head-to-tail homodimer with an interaction interface between Macro and PLpro. However, PDCoV Macro-Ubl2-PLpro mainly exists as a monomer in solution. Then, we conducted fluorescent resonance energy transfer-based protease assays and found that PDCoV PLpro can cleave a peptide by mimicking the cognate nsp2/nsp3 cleavage site in peptide substrates and exhibits deubiquitinating and de-interferon stimulated gene(deISGylating) activities by hydrolysing ubiquitin-7-amino-4-methylcoumarin (Ub-AMC) and ISG15-AMC substrates. Moreover, the deletion of Macro or Macro-Ubl2 decreased the enzyme activity of PLpro, indicating that Macro and Ubl2 play important roles in maintaining the stability of the PLpro domain. Two active sites of PLpro, Cys260 and His398, were determined; unexpectedly, the conserved site Asp412 was not the third active site. Furthermore, the motif “NGYDT” (amino acids 409–413) was important for stabilizing the enzyme activity of PLpro, and the N409A mutant significantly decreased the enzyme activity of PLpro. These results provide novel insights into the replication mechanism of CoV and new clues for future drug design.

## Introduction

Coronaviruses (CoVs) are enveloped, positive-sense single-stranded RNA (+ssRNA) viruses belonging to the family *Coronaviridae* of the order *Nidovirales* and have the largest genomes (26–32 kb) among known RNA viruses [1]. CoVs are divided into four genera: *alpha-*, *beta-*, *gamma-* and *delta-coronavirus* (α-CoV, β-CoV, γ-CoV, and δ-CoV, respectively). CoVs can infect many species [2,3]; CoVs that infect humans are mainly from α-CoV and β-CoV. Human CoV 229E (HCoV-229E) and human CoV NL63 (HCoV-NL63) belong to α-CoV, severe acute respiratory syndrome CoV (SARS-CoV), Middle East respiratory syndrome (MERS-CoV) and emerging severe acute respiratory syndrome coronavirus 2 (SARS-CoV-2) belong to β-CoV [4]. CoVs infecting animals are found in four genera, including feline infectious peritonitis virus (FIPV), transmissible gastroenteritis virus (TGEV), porcine epidemic diarrhea coronavirus (PEDV), murine hepatitis virus (MHV), infectious bronchitis virus (IBV) and porcine delta-coronavirus (PDCoV) [2,5]. Although CoVs cause severe threats to the health of humans and animals, effective commercially available vaccines or drugs are not available to treat CoVs except for the commercial vaccine against FIPV [6].

The 5’-terminal two-thirds of the CoV genome include two open reading frames (ORF1a and ORF1b) that encode two viral replicase polyproteins (ppla and pplab) [7,8]. The two polyproteins are hydrolysed into 10–16 nonstructural proteins (nsps) by papain-like protease (PLpro of nsp3) and 3C-like protease (3CLpro or nsp5) and form mature functional nsps through posttranslational modification [9]. Then, the mature nsps together form the replication/transcription complex (RTC), which participates in the formation of double-membrane vesicles (DMVs) on the endoplasmic reticulum (ER) [10]. Nsp3 is essential for the formation of the RTC, and it functions as a scaffold protein by interacting with itself and other proteins (including viral nsps and host proteins) [5,10]. As the largest multidomain protein in CoVs, although some domains are absent or repeated among different CoV genera, nsp3always contains the following conserved domains: ubiquitin-like domain 1 (Ubl1), hypervariable region (HVR), macrodomain (Macro, also named the ADRP or X domain), ubiquitin-like domain 2 (Ubl2), papain-like protease 2 (PLP2), ectodomain, Y1 and CoV-Y domains and two transmembrane regions (TMs). PDCoV contains 15 nsps, but the structures of most PDCoV nsps have not been reported so far except for the structure of nsp9 [11].

Macrodomains are ancient, conserved domains that occur widely across various organisms, such as bacteria, archaea and eukaryotes [12], and even in hepatitis E virus (HEV) and CoVs [13,14]. Macro is named after the nonhistone domain of the histone macroH2A. Affinity for adenosine diphosphate ribose (ADPr) or poly-ADP-ribose is a well-known characteristic of Macro, and in some cases, it also shows ADP-ribose 1″-monophosphatase (ADRP) activity [15]; however, the biological significance of this enzyme activity remains unclear [16–19]. According to previous reports, CoVs Macros may mediate resistance to the antiviral interferon response, and the SARS-CoV Macro exhibits the ability to inhibit the expression of innate immunity-related genes [3,18]. These possible biological functions are consistent with the functions of SARS-CoV PLpro and HCoV-229E PLP2, revealing that synergy may exist between the two domains. Excitingly, a recent report indicates that the interplay between Macro and PLpro may affect viral replication and pathogenesis [20]. Several unliganded or liganded structures of CoV Macro have been reported to date, including SARS-CoV, SARS-CoV-2, MERS-CoV, HCoV-229E, IBV and FCoV Macros [16,21–23]. Based on these studies, Macro can be a drug target, and the different domains of nps3 may exhibit cooperative interactions.

PLpro is responsible for the hydrolytic release of nsp1/2 -nsp4; in addition to hydrolytic activity, PLpro also possesses deubiquitinating and deISGylating activities [24–27]. Lys48-, K63- and linear ubiquitin are three types of polyubiquitination that are associated with immune signalling pathways, and PLPs remove the K48-Ub and K63-Ub from target proteins by recognizing the LRGG motif [28–30]. Interferon-stimulated gene 15 protein is a ubiquitin-like molecule that conjugates to the target protein, such as RIG-I, JAK1, STAT1, PKR and MxA. PLPs have the ability to remove conjugated ISG15 cellular proteins [30–32]. Thus, the deubiquitinating and deISGylating activities of PLPS are considered two pathways by which CoVs escape the immune response. PLPs block the IFN regulatory factor 3 (IRF3) induced IFN-β and TNFα-mediated NF-κB activation [33].

As previous reports, PLPs contain the classic Cys–His–Asp triad. Interestingly, HCoV-229E PLP1 exhibits a Cys–His dyad [34], while the homologue PLpro of equine arteritis virus (EAV) comprises a Cys–His–Asn triad [35]. Ubl2 exists in CoVs and among host ubiquitin-specific proteases (USPs), which regulate the catalytic activity of proteases [36,37].

At present, the structure and function of PDCoV PLpro have not been reported. Here, we first report the structure of PDCoV Macro-Ubl2-PLpro from δ-CoV with 2.5 Å resolution. The structure contains several important structural features, including the arrangement of domains from Macro to PLpro in nsp3, the interaction between Macro and PLpro in the crystal homodimer, and the catalytic core of PDCoV PLpro. We performed *in vitro* assays using substrates representing viral and cellular targets to characterize the enzyme activities of the purified protease. Our results show that PDCoV PLpro has hydrolytic activity, deubiquitinating and deISGylating activities, and Ubl2 plays important roles in stabilizing the PLpro enzyme activities.

## Materials and methods

### Plasmid construction

The sequence encoding the PDCoV Macro-Ubl2-PLpro domain (residues 939–1384 of the polyprotein ppla from PDCoV strain CHN-HB-2014, GenBank accession number KP757891.1) was amplified by PCR from the parental plasmid pCAGGS-HA-nsp3, which was kindly provided by Professor Xiao and cloned into the pET-42b vector. Macro-Ubl2-PLpro mutants (C260A, H398A, D412A, NGYDT409-413AAAAA, N409A, G410A, Y411A, and T413A) and truncations (Ubl2-PLpro, PLpro) were also cloned into pET-42b with C-terminal His_6_ tags. All of the recombinant plasmids were sequenced.

### Protein expression and purification

The recombinant plasmids were transformed into *E. coli* BL21 (DE3) cells for expression. Cultures were grown in LB medium at 37°C until the optical density at 600 nm (OD600) reached 0.6–0.8, induced with 1 mM isopropyl-β-D-thiogalactopyranoside (IPTG), and incubated with shaking overnight at 27°C. To solve the phase problem, selenomethionine (Se-Met)-labelled PDCoV Macro-Ubl2-PLpro was expressed in *E. coli* BL21 (DE3) cells according the instructions of the M9 Se-Met High-Yield Growth Media Kit (Medicion, China), and cultured with 50 µg/mL kanamycin, M9 salt medium, 15 mineral supplements, vitamins (thiamine, vitamin B12), 0.5% glycerol at 37°C until reaching the OD600 of 1.2. Then, the amino acid mixture was added (lysine, phenylalanine, threonine, isoleucine, leucine, valine, and selenomethionine), 15 min later, IPTG was added at a final concentration of 1 mM and cells grown at 37°C for 5 h.

For protein purification, cells were pelleted by centrifugation at 8,000 rpm (5 min at 4°C) from 1 L of culture, resuspended in 50 mL of phosphate-buffered saline (PBS) and lysed using an ultrahigh-pressure cell disrupter (ATS Engineering Inc.). After centrifugation at 8,000 rpm for 30 min, the supernatant was filtered through a 0.45-µm-pore-size filter and loaded onto a His Trap HP column (GE Healthcare). The target protein was eluted with a linear gradient between the binding buffer (20 mM Tris-HCl [pH 7.4] and 500 mM NaCl) and elution buffer (20 mM Tris-HCl [pH 7.4], 500 mM NaCl and 500 mM imidazole). The protein was further purified using a Superdex200 gel filtration column (GE Healthcare) equilibrated with buffer (20 mM Tris-HCl [pH 7.4] and 200 mM NaCl). For crystallization, the purified protein was concentrated to 12 mg/mL using a 30-kDa-molecular-mass-cutoff centrifuge concentrator, and the concentration was determined by measuring the absorbance at 280 nm with a Nano Drop spectrophotometer (Thermo Scientific). The protein sample was flash-frozen with liquid nitrogen and stored at −80°C. Notably, to avoid unexpected degradation, all of the purification procedures should be performed at 4°C.

### Crystallization, data collection, and structure determination

Crystallization screening for native Macro-Ubl2-PLpro at a concentration of 12 mg/mL was performed at 20°C using hanging drop vapour diffusion with 96-well plates. Crystals were obtained from a solution containing 0.1 M imidazole and 12% polyethylene glycol (PEG) 20,000. Further optimization of the crystallization conditions was performed with 24-well plates through vapour diffusion in sitting drops consisting of a 1 µL drop of 12 mg/mL protein, 0.1 M imidazole, and 10–16% PEG 20,000. The crystals grew overnight and were cryoprotected by the addition of 20% ethylene glycol and flash-cooled in liquid nitrogen. The selenomethionine derivative Macro-Ubl2-PLpro was crystallized under similar conditions. Single-wavelength X-ray diffraction data were collected from single crystals at the BL17U1 beam line (wavelength=0.97910 Å, temperature=100 K) of the Shanghai Synchrotron Radiation Facility (SSRF). All data were processed with HKL-3000 software [38], and the resulting statistics are listed in Table 1. The initial structure was solved using the single-wavelength anomalous dispersion (SAD) method from the Se-Met derivative and molecular replacement with PHASER [39]. All five potential selenium atoms in the Macro-Ubl2-PLpro monomer were located, and the initial phases were calculated using the program AutoSol from the PHENIX software suite [40]. Manual model rebuilding was performed using Coot [41]. Refinement was carried out using the program PHENIX. Structural figures were drawn using the program PyMOL [42]. The amino acid sequences of CoV Macro and Ubl2-PLpro were aligned using ClustalW2 [43] and visualized with the ESPript 3 server (http://espript.ibcp.fr) [44].

**Table 1.** Data collection and refinement statistics.

	Value
Parameter	Mac-Ubl2-PLpro
**Data collection**	
Space group	C121
Cell dimensions	
*a*, *b*, *c* (Å)	139.92, 71.35, 64.43
*α*, *β*, *γ* (°)	90.00, 92.26, 90.00
Resolution (Å)	39.02-2.46
Completeness (%)	98.4
*R_merge_*^a^	2.483(0.088)
*I/σ*(*I)*	37(2.83)
Redundancy	16.2(12.7)
**Refinement**	
Resolution (Å)	39.2-2.46
No. of reflections	22,928
*R*_work_/*R*_free_^b^	20.81/25.94
**No. of atoms**	
Protein	3280
Ligand/ion	1
Water	26
**RMSD**	
Bond lengths (Å)	0.009
Bond angles (°)	1.027
**Ramachandran analysis**	
Favored (%)	93.62
Allowed (%)	5.67
Outliers (%)	0.71

Notes: The highest-resolution values are indicated in parentheses.

^a^ *R*_merge_ = ∑∑∣*I_i_*–<*I*>∣/∑∑*I_i_*; where *I_i_* is the intensity measurement of reflection *h*, and <*I*> is the average intensity from multiple observations.

^b^ *R*_work_ = ∑||*F_o_*|–|*F_c_*|| /∑|*F_o_*|; where *F_o_* and *F_c_* are the observed and calculated structure factors, respectively. *R*_free_ is equivalent to *R*_work_, but 5% of the measured reflections have been excluded from the refinement and set aside for cross-validation.

### Small angle X-ray scattering analysis

SAXS data were collected at the BL19U2 beamline of the Shanghai Synchrotron Radiation Facility (SSRF) at room temperature. For the SAXS measurement, 25 µM PDCoV Macro-Ubl2-PLpro was prepared in buffer (20 mM Tris-HCl [pH 7.4] and 200 mM NaCl). For each measurement, 20 consecutive frames with 1-sec exposure were recorded and averaged, with difference between the first and the last frames. The background scattering was recorded for the matching buffer and was subtracted from the protein scattering data. The data were visualized and analysed using the software package ATSAS [45]. The theoretical SAXS profiles of monomer and dimer crystal structures were calculated with the software CRYSOL.

### Steady-state kinetic analysis

Assays to determine the peptide cleavage of a fluorescent resonance energy transfer (FRET) substrate, DABCYL-PGFKAGSDELFI-(E-EDANS)-amide (DABCYL, 4-{[(4-dimethylamino) phenyl]azo benzoic acid; EDANS, 5-[(2-aminoethyl) amino] naphthalene-1-sulfonic acid) (GenScript, Nanjing, China), were performed using different substrate concentrations (10–50 µM) and 2 µM purified PDCoV Macro-Ubl2-PLpro. Assays were performed in 20 mM Tris-HCl, pH 7.5, 200 mM NaCl at 30°C with a 96-well microplate using the multimode reader platform (Tecan). The rate of substrate hydrolysis was determined by monitoring the fluorescence as a function of time (excitation λ, 336 nm; emission λ 490 nm) and calculated from the linear part of the curves. Since no saturation was observed in the plot of initial velocities versus substrate concentrations, data points were fit to the equation *v*/[*E*]_Total_=*k*_app_[*S*] to determine the pseudo-ﬁrst-order rate constant *k*_app_ assuming that [S]<<*K_m_*; *v* is the initial velocity (µM/min); [*E*] and [*S*] are the concentrations of enzyme and peptide substrate (µM), respectively; *k*_app_ is the pseudo-first-order rate constant; and for this equation, *k*_app_ approximates *k*_cat_/*K*_m_. The same measurements were repeated for the other purified proteins. Fluorescence intensity was converted to the amount of hydrolysed substrate using a standard curve drawn from the fluorescence measurements of well-defined concentrations of DABCYL-PGFKAG and SDELFI (E-EDANS)-amide peptides in a 1:1 ratio. Data processing and image generation were performed using GraphPad Prism 7.0 software (GraphPad).

### *In vitro* assay of deubiquitinating and DeISGylating activities

Assays to determine the deubiquitinating and deISGylating activities of the purified protein were performed using the fluorogenic substrate human Ub-7-amino-4-trifluoro-methylcoumarin (Ub-AMC) and ISG15-AMC (Boston-Biochem). The reaction was conducted in the presence of a solution containing 20 mM Tris-HCl, pH 7.4, and 200 mM NaCl at 30°C. Assays were performed with a 96-well microplate using the multimode reader platform (Tecan). The reaction mixtures contained different substrate concentrations (0.1–0.5 µM) and 1 µM protein. Fluorescence was monitored continuously (excitation λ, 335 nm; emission λ 445 nm). The initial velocities were plotted against the fluorogenic substrate concentration and fitted to the equation presented above to determine the pseudo-first-order rate constant. The standard curve was generated by measuring the fluorescence of AMC dissolved in the reaction buffer at different concentrations (0.1–0.6 µM). Data processing and image generation were performed using GraphPad Prism 7.0 software (GraphPad).

### DUB assay in lysates

HeLa cells were cultured with medium containing penicillin/streptomycin and 10% FBS at 37°C in the presence of 5% CO_2_. Cells were

stimulated with 100 ng/mL human IFN-β for 48 h, and then incubated with 10 µM MG132 (Sigma) for 4 h before harvest. HeLa cells were treated with 10 µM MG132 for 30 min and then stimulated with 10 ng/mL TNF-α for 15 min at 37°C. Cells were harvested and treated as previously described [46]. The 20 µL reaction volumes contained 200 nM DUBs and 10 µg of total lysate with 25 mM DTT; reactions were terminated by heating with SDS loading buffer and analysed using SDS-PAGE and western blotting with the indicated antibodies. Antibodies used in this study were anti-ubiquitin (P4D1, Santa Cruz Biotech), anti-ISG15 (Bioswamp), anti-His (Proteintech), anti-K48-ubiquitin (Boster), goat anti-rabbit secondary antibodies (Proteintech), and goat anti-mouse IgG light-chain secondary antibodies (Proteintech). Images were captured with an Amersham Imager 600 (GE Healthcare) imaging system.

## Results

### Overall structure of PDCoV Macro-Ubl2-PLpro

**Figure 1.**
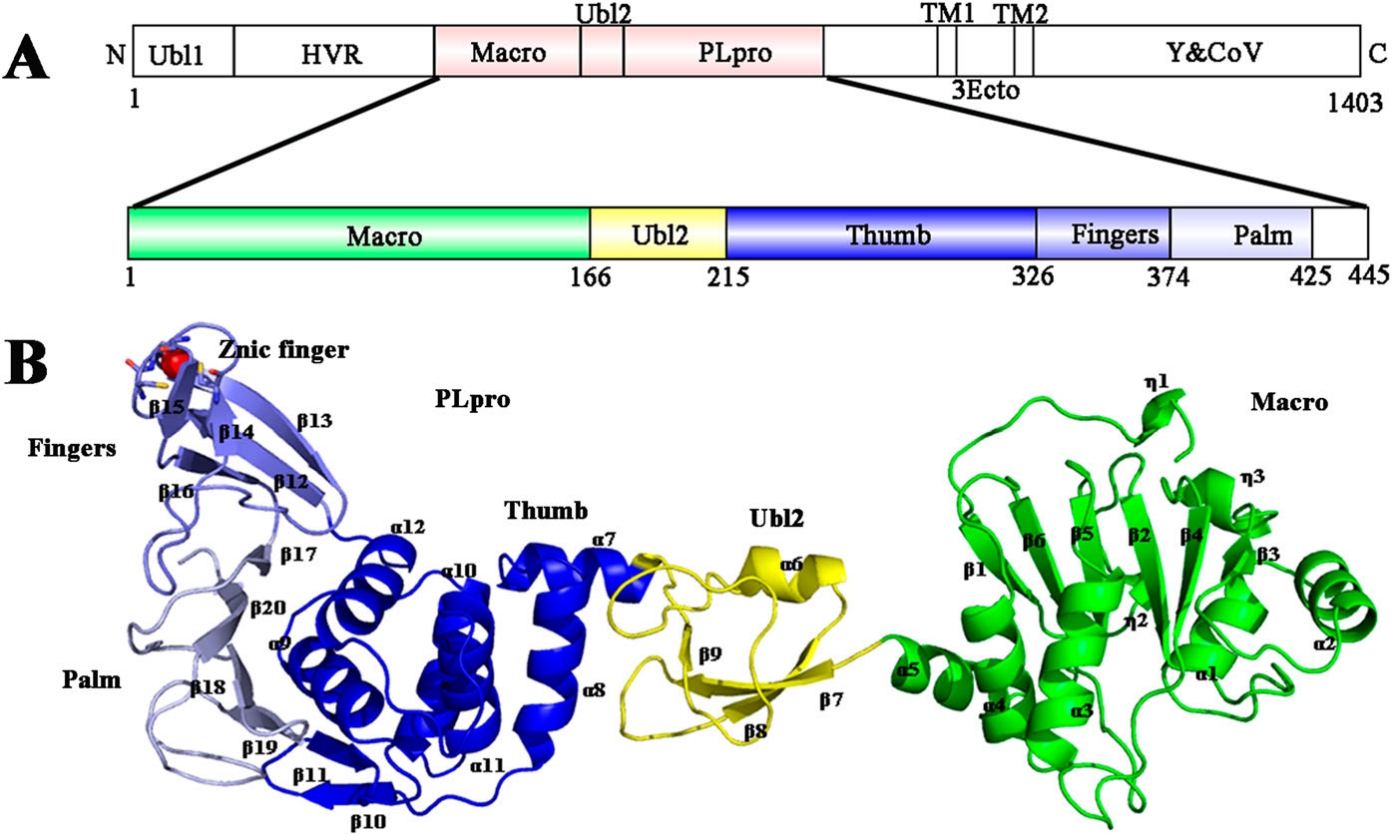
Organization of the PDCoV nsp3 genome and the overall structure of PDCoV Macro-Ubl2-PLpro. (A) Arrangement of the different functional subdomains of PDCoV nsp3: Ubl, ubiquitin-like domain 1; HVR, hypervariable region; Macro, macrodomain; Ubl2, ubiquitin-like domain 2; PLpro, papain-like protease domain; TM1 and TM2, transmembrane regions 1 and 2; 3Ecto, nsp3 ectodomain; Y1 and CoV-Y, Y1 and CoV-Y domain. The constructed regions used in the assay are highlighted in pink; the organization of the PDCoV Macro-Ubl2-PLpro domains was inferred from the crystal structure. (B) Ribbon representations of the subunits of the PDCoV Macro-Ubl2-PLpro structure. Secondary structures (helices, strands and loops) are marked; α-helices are labelled from α1 to α12, β-stands are labelled from β1 to β20, and Zn^2+^ ions are shown as a red sphere. In A and B, the domains are coloured in the same manner.

PDCoV Macro-Ubl2-PLpro (polyprotein residues 939 to 1384) was produced as a soluble protein in *E. coli* to investigate the structure of PDCoV nsp3*.* The final purified protein was a 445-amino acid protein (49.6 kDa) (Figure S1A) with an additional His_6_ tag at the C-terminus. The crystal structure of Macro-Ubl2-PLpro was determined using the single-wavelength anomalous diffraction (SAD) method and the molecular replacement program PHASER, which was refined to a resolution of 2.5 Å, with final *R*-values of *R*_work_ = 20.81% and *R*_free_ = 25.94%. The crystal belongs to the space group C121, each asymmetric unit contains two subunits, and the solvent content value is 62.3%. The final PDCoV Macro-Ubl2-PLpro structural model had observable electron density for residues 1–425 (Figure 1(A)). The details of phasing and refinement were provided in Table 1.

The Macro-Ubl2-PLpro monomer consists of three distinct domains: Macro, Ubl2, and PLpro (Figure 1(B)). At the flanking N-terminus of the crystal structure, Macro adopts a conserved three-layered α-β-α sandwich fold, similar to the macroH2A variant of human histone 2A [47]. Amino acids (aa 166-215) fold into a ubiquitin-like 2 domain, which lies in front of PLpro and is well separated from PLpro and Macro. The thumb, palm and finger domains form the catalytic core of PLpro, which has an extended right-hand architecture (Figure 1(B)).

In the asymmetric unit, two of the subunits form the head-to-tail homodimer, with a total area of 807.3 Å^2^ buried in the interface (Figure S2). Although a number of polar/electrostatic interactions are present in the interface, the majority of the interactions occur via the formation of a hydrophobic network involving residues Thr60, Ser63, Val64, Ser68, Asp74, Phe76 from Macro and Ala348, Arg349, Thr382, Ala384, Phe394, Ala396, Ala397, Asn416 from PLpro (Figure S2B). However, PDCoV Macro-Ubl2-PLpro primarily exists as a monomer when analysed using gel-filtration chromatography (Figure S1A). The SAXS profile calculated based on the monomer crystal structure is consistent with that in solution, while the profile calculated based on the dimer crystal structure is inconsistent, indicating that the protein is monomer in solution. In addition, the molecular weight measured with SAXS was 47.7 kDa, and the value of Rg was 36.7 Å, which is also consistent with the monomer structure (the Rg of the monomer structure is 37.4 Å) (Figure S1B).

### Relatively conserved Macro of PDCoV

**Figure 2.**
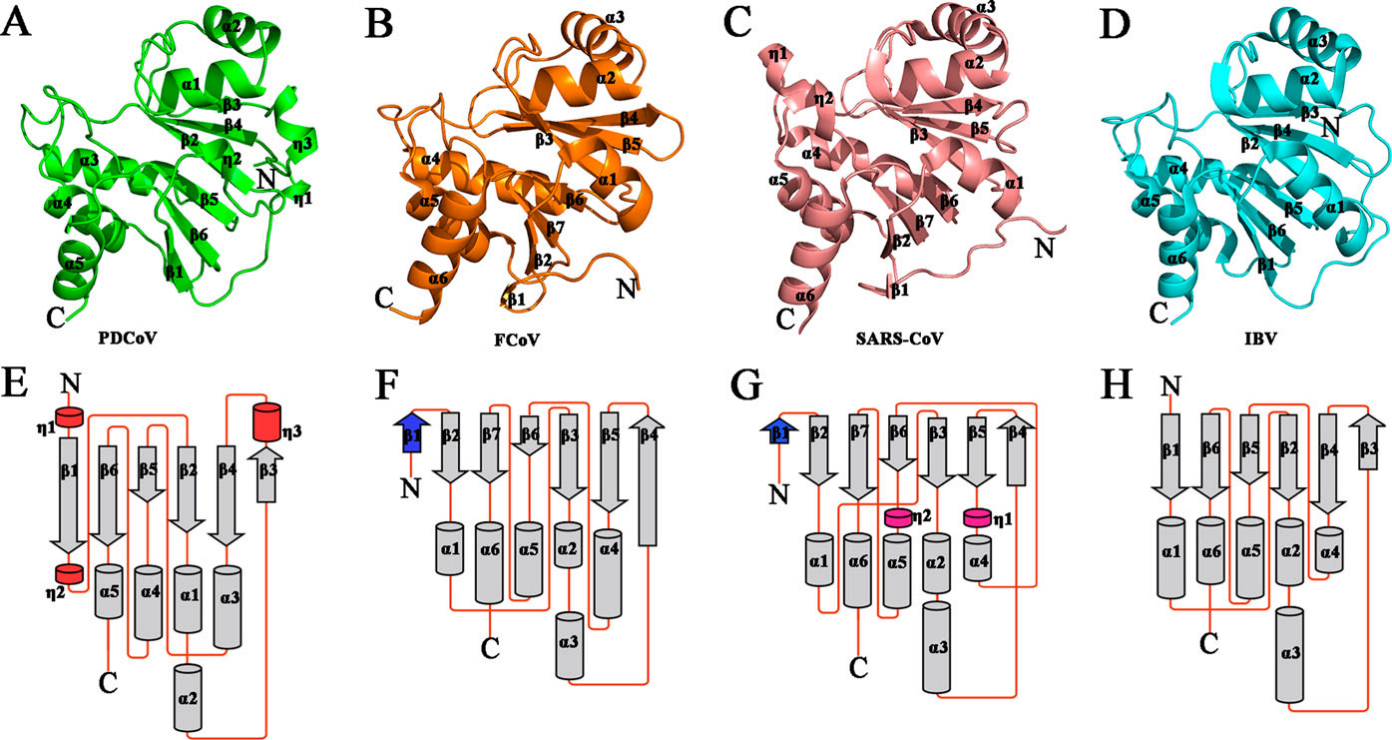
Structural comparison and topology diagram of Macros among the four CoV genera. (A)–(D) Detailed structures of PDCoV Macro from δ-CoV (green), FCoV Macro from α-CoV (orange), SARS-CoV Macro from β-CoV (salmon), and IBV Macro from γ-CoV (cyan). From A to D, secondary structures (helices, strands, and loops) are marked. (E)–(G) Topology diagrams of PDCoV Macro from δ-CoV, FCoV Macro from α-CoV (orange), SARS-CoV Macro from β-CoV (salmon), and IBV Macro from γ-CoV (cyan). From E to G, the β-strands are shown as arrows, and the α-helices and 310-helices (η) are drawn as cylinders. Coronavirus macrodomains have a very similar topology. The differences in the topologies are coloured (red in PDCoV, blue in FCOV and SARS-CoV, and magenta in SARS-CoV). The PBD IDs of FCoV, SARS-CoV and IBV Macros are 3ETI, 2ACF and 3EWP, respectively.

PDCoV Macro adopts the same conserved three-layered α-β-α sandwich fold as Macro from the other three CoV genera. The sandwiched six-stranded β-sheet is surrounded by η2-α1-α2-η3 packing on one side and α3-α4-α5 packing on the other side, and a short 3_10_- helix is present at the C-terminus of the structure (Figure 2). The central β- sheet with six β-strands in PDCoV Macro is in line with IBV; unlike the structure observed in other CoV genera, a first strand is not observed at the N-terminus of the Macro of PDCoV. In the Macro of PDCoV, two short 3_10_-helices are detected that are not observed in the other three CoV genera, the helix η1 occurring at the N-terminus and the other helix η3 lying between β3 and β4 (Figure 2(A,E)). The Macros of FCoV, SARS-CoV and IBV contain three α-helices (α1, α2, α3 and α4, α5, α6) that pack on each side of the sandwiched six β-strands (Figure 2(B–D and F–H)); a short 3_10_-helix (*η*2) occupies the position of the third α-helix (α3) of PDCoV Macro (Figure 2(A,E)), and the structural features of PDCoV Macro are consistent with the macroH2A-like domain [15].

A deep solvent-exposed cleft on the protein surface in the PDCoV Macro is similar to that of FCOV, SARS-CoV, and IBV Macros. The distribution of the electrostatic potential on the PDCoV Macro and ligand-bound forms of FCoV, SARS-CoV and IBV Macros are shown as surface representations (Figure S3A–D). Although the putative active site cleft and putative active sites are similar to that of other CoVs Macros (Figure S3), attempts at soaking and cocrystallization with ADP-ribose failed to yield crystal compounds. The putative active sites are shown in the sequence alignment, and four stretches of amino acid residues involve the binding with ADP-ribose (Figure S3E). The first motif is “GEX” in PDCoV Macro, instead of the conserved “GDE” among other CoVs. The second motif is “XVNPAN,” although putative active sites Asn33 and Asn36 are conserved, the conserved Ala between them is replaced by Pro in PDCoV Macro, which may influence the interaction between Macro and ADP-ribose. The third motif is the Gly-rich region (“NGGGXA”), in which Asn41 occupies the position of the conserved positively charged amino acid His, possibly decreasing the ADP-ribose binding affinity. The final conserved motif is “GIF,” which includes Phe127 and may participate in the interaction between ADP-ribose and Macro (Figure S3E).

### Subtle but important differences between PLpro of PDCoV and other PLPs

**Figure 3.**
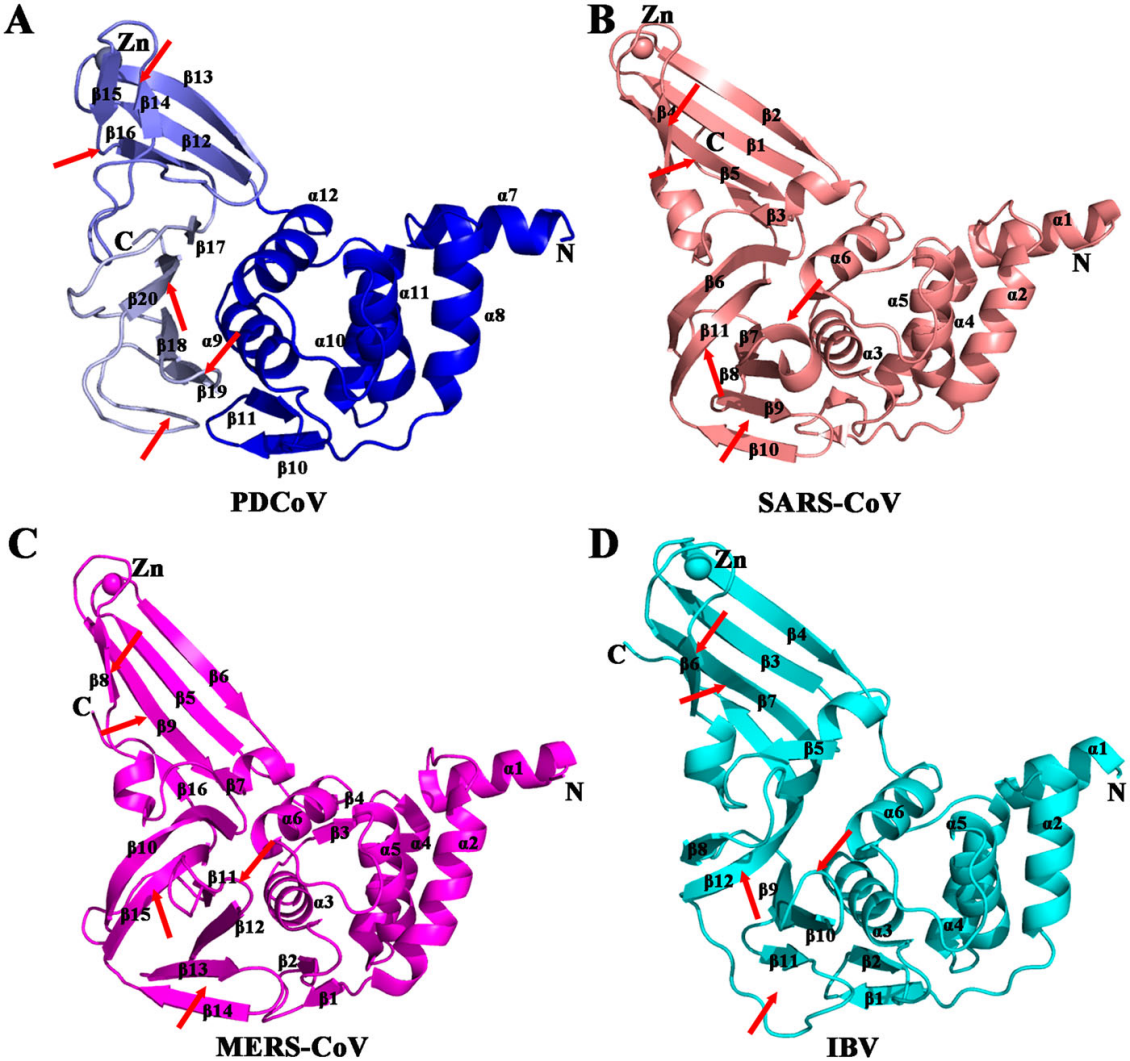
Structural comparison of PLPs among four CoVs. (A) Detailed structure of PDCoV PLpro from δ-CoV (blue). (B) Structure of SARS-CoV PLpro from β-CoV (PDB ID: 2FE8, salmon[27]). (C) Structure of MERS-CoV PLpro from β-CoV (PDB ID: 4RNA, magenta [60]). (D) Structure of IBV PLpro from γ-CoV (PDB ID: 4X2Z, cyan [24]). From A to D, the secondary structures (helices, strands, and loops) are marked; Zn^2+^ ions are shown as spheres. Regions that show significant differences among genera are indicated by arrows.

The comparison of the entire PDCoV PLpro monomer with other CoVs PLPs structures in the Protein Data Bank (PDB) revealed several subtle but important differences. The fold of PDCoV PLpro is similar to the folds of SARS-CoV, MERS-CoV and IBV PLPs, and the thumb domain is highly conserved among CoVs and composes six *α*-helices and two short antiparallel β-strands, the β-sheet between helices α8 and α9 that are arranged in a β-hairpin. Following the thumb domain is the finger domain (aa 326-373) which contains three long stands (β12, β13 and β16) and two short antiparallel stands (β14 and β15). At the tip of the finger domain, four cysteines (Cys334, Cys335, Cys360 and Cys363) from two β-hairpins that coordinate a zinc ion with a tetrahedral structure. The palm domain is primarily composed of four antiparallel strands (β17-β20) (Figures 1 and 3(A)).

Two structural differences occur in the finger domains. First, the starting point and the overall direction of the β14 N-terminus are obviously twisted compared to those of other CoV PLPs (Figure 3). The second is that two disjoined β-strands (β15 and β16) are replaced by one β-strand in SARS-CoV, MERS-CoV and IBV PLPs (Figure 3(B–D)). Thus, the finger domain of PDCoV PLpro protrudes out from the structure to a lesser extent than the finger domains of other CoV PLPs.

Three important differences occur in the palm domain. Strands β17 and β20 of PDCoV PLpro are obviously shorter than that of other CoVs PLPs. In particular, the loop connecting β18 and β19 is likewise shorter than that of other CoV PLPs (Figure 3 and Figure S4). This loop is first described as “blocking loop 2 (BL2)” in USP14, and it participates in regulating the USP14 deubiquitinating activity through affecting the binding with the substrates [28,48]. The short BL2 in PDCoV PLpro causes the space for substrate binding to be much larger than the corresponding spaces of USP14 and other CoV PLPs. Following β19, a long loop connects β19 and β20, with no additional rigid structure between them (Figure 3(A)). This feature is quite inconsistent with the previously reported PLPs: instead of one long loop, two β-strands occupy this position in MERS-CoV and SARS-CoV PLPs, and at least one β-strand occupies this position in IBV PLpro (Figure 3(B–D)). Interestingly, these additional β-strands slope into the active sites, which are housed in a solvent-exposed cleft between the thumb and palm domains due to the missing β-strand and the short β20 in the palm domain of PDCoV PLpro (Figure 3(A)). The orientation of the loop is shifted from underneath β19 to the side of β19; as a result, the solvent-exposed cleft becomes larger than that of other CoV PLPs (Figure 3).

### Significantly offset Ubl2

**Figure 4.**
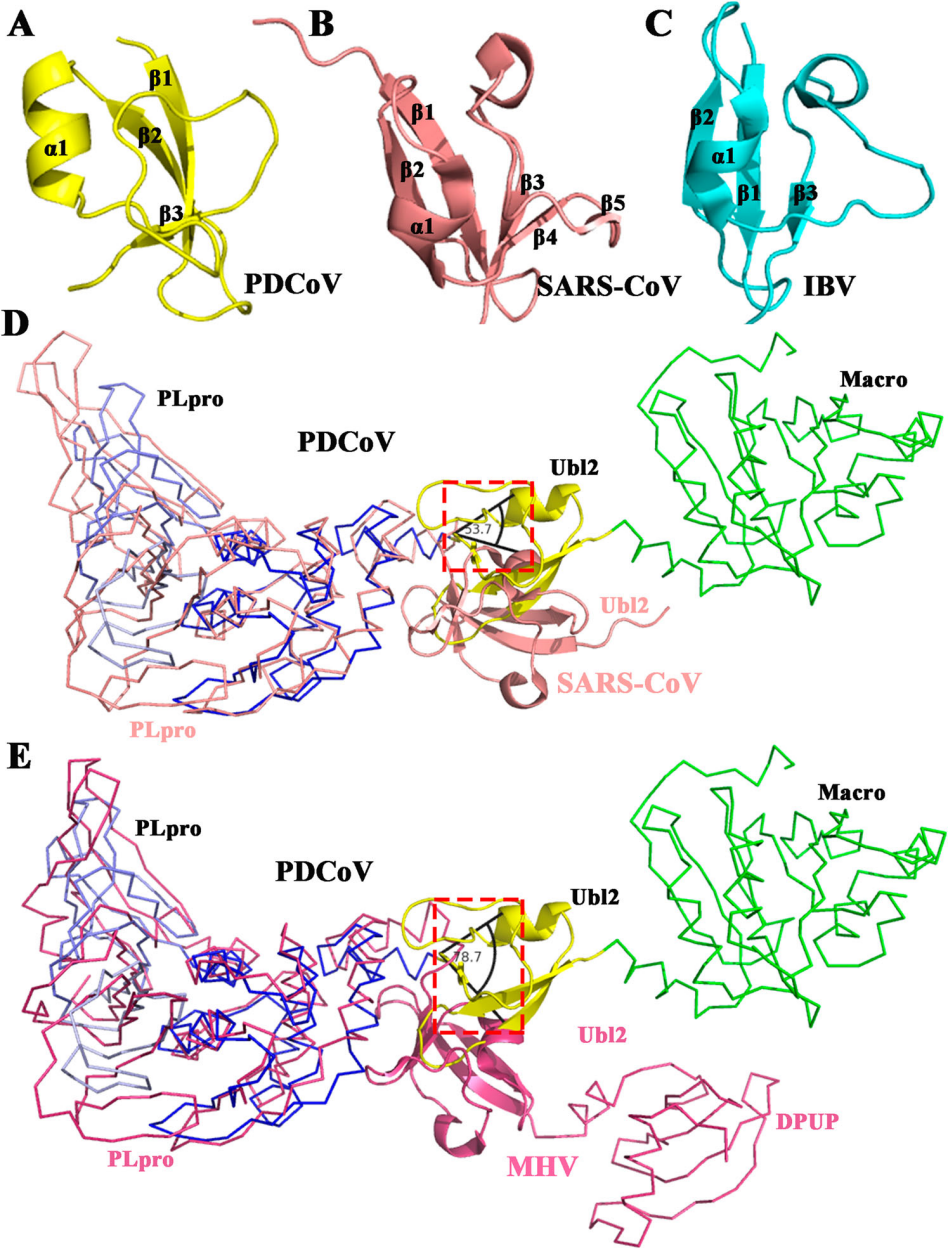
Structural comparison of Ubl2 from three CoV genera. (A) Detailed structure of PDCoV Ubl2 from δ-CoV (yellow). (B) Structure of SARS-CoV PLpro from β-CoV (PDB ID: 2fe8, salmon). (C) Structure of IBV PLpro from γ-CoV (PDB ID: 4×2z, cyan). From A to C, secondary structures (helices, strands and loops) are marked. (D) and (E) Crystal structures of SARS-CoV Ubl2-PLpro (PDB ID: 2fe8, salmon) and MHV DPUP-Ubl2-PLP2 (PDB ID: 4ypt, warm pink) are superimposed over the structure of PDCoV Macro-Ubl2-PLpro (in multiple colours). Macro of PDCoV, the DPUP (domain preceding Ubl2 and PLP2) of MHV and the PLPs from PDCoV and SARS-CoV are shown as ribbons, and MHV, Ubl2 from PDCoV, SARS-CoV and MHV are shown as cartoons. The deviation angles of Ubl2 are within the black lines.

PDCoV Ubl2 consists of a β-grasp fold that is similar to the β-grasp folds of ubiquitin and the Ubl2 of SARS-CoV and IBV, while the Ubl2 of PDCoV is significantly smaller than SARS-CoV Ubl2 (Figure 4(A–C)). No structures of multifunctional domains of nsp3 are available, except for MHV DPUP-Ubl2-PLP2, so structural superpositions of PDCoV Mac-Ubl2-PLpro, SARS-CoV Ubl2-PLpro and MHV DPUP-Ubl2-PLpro are performed. The structural superpositions show that the orientation of PDCoV Ubl2 is reversed compared with SARS-CoV and MHV Ubl2 (Figure 4(D,E)), even if the DPUP domain exists at the N-terminus of the MHV tandem structure, the orientation of MHV Ubl2 is not reversed (Figure 4(E)), therefore excluding the traction of Macro.

### Peptide-based *in vitro* cleavage assay

PLpro is well known for its hydrolytic activity, therefore, we firstly characterized purified PDCoV Macro-Ubl2-PLpro to determine its proteolytic activity toward peptides mimicking viral substrate. The substrate is a synthetic peptide derived from the nsp1/nsp2 cleavage site of PDCoV, which consists of the 12 amino acids PGFKAGSDELFI. DABCYL and EDANS moieties are fused to the N and C termini of the peptide. Peptide hydrolysis in the FRET-based kinetic assay was detected by measuring the increased fluorescence intensity at 490 nm, and the released EDANS is no longer quenched by the DABCYL group.

No saturation was observed in the plot of initial velocities against substrate concentrations, in order to determine the pseudo-first-order rate constant (*k*_app_, approximates *k*_cat_/*K*_m_) for PDCoV PLpro hydrolytic activity, the enzyme activity was linearly proportional to the concentration of the peptide substrate. The *k*_app_ for PDCoV Macro-Ubl2-PLpro hydrolysis of the short synthetic substrates, which was calculated from initial reaction rates obtained at different peptide concentrations, was determined to be 0.66±0.03 min^−1^mM^−1^ (Table 2). This value is similar to the TGEV PLP1 *k*_app_ obtained for the hydrolysis of the substrate Dabcyl-MYNKMGGGDKTVSF(E-EDANS)-amide [26].

**Table 2.** Apparent *k*_cat_/*K*_m_ (*k*_app_) values for PDCoV PLpro.

PDCoV	Peptide hydrolysis	Deubiquitination	DeISGylation
	*k*_app_^a^(min^−1^ mM^−1^)^b^	*k*_app_ (min^−1^ mM^−1^)^c^	*k*_app_ (min^−1^ mM^−1^)^d^
Macro-Ubl2-PLpro	0.66 ± 0.03	9.5 ± 0.8	12.5 ± 1.5

Note: Values are presented as standard deviations (±SD) with 95% confidence interval (*n* = 3, independent experiments).

^a^ For non-saturating substrates, *k*_app_ is calculated to approximate *k*_cat_/*K*_m_.

^b^ Substrate for peptide hydrolysis: DABCYL-PGFKAGSDELFI(E-EDANS)-amide.

^c^ Substrate for deubiquitination: ubiquitin-AMC.

^d^ Substrate for deISGylation: ISG15-AMC.

### *In vitro* deubiquitinating and DeISGylating activities of PDCoV Macro-Ubl2-PLpro

After testing the hydrolytic activity, we used the commercially available fluorescent substrate Ub-AMC and ISG15-AMC to test the deubiquitinating and deISGylating activities of PDCoV Macro-Ubl2-PLpro. PDCoV Macro-Ubl2-PLpro cleaves the bond between ubiquitin/ISG15 and AMC, resulting in the release of the fluorescent dye and an increase in the fluorescence intensity. As with the fluorescent peptide, no saturation was observed with the ubiquitin/ISG15 substrate at the concentrations assayed. The pseudo-first-order rate constant of PDCoV Macro-Ubl2-PLpro deubiquitinating activity was determined to be 9.5±0.8** **min^−1^mM^−1^, which was significantly lower than that of SARS-CoV PLpro (4480** **min^−1^mM^−1^) [49], and TGEV PLP1 (74** **min^−1 ^mM^−1^) [26]. Interestingly, the *k*_app_ rate constant of PDCoV Macro-Ubl2-PLpro was similar to the *k*_app_ of USP7 (13** **min^−1 ^mM^−1^) [50]. Similarity, PDCoV Macro-Ubl2-PLpro *k*_app_ for cleaving ISG-AMC substrate was determined to be 12.8 ± 1.5** **min^−1 ^mM^−1^. Thus, PDCoV Macro-Ubl2-PLpro showed a stronger preference for ubiquitin and ISG15 substrates than the FRET peptide (Table 2).

SARS-CoV PLpro and MERS PLpro remove the Lys48-linked ubiquitin chain from conjugated substrates, and the SARS-CoV PLpro preferentially recognizes and releases diUb-Lys48, in contrast to MERS-CoV PLpro [46]. In our study, we tested the ability of PDCoV Macro-Ubl2-PLpro to hydrolyse K48-linked poly-ubiquitylated conjugates and ISGylated conjugates of cellular proteins. PDCoV PLpro cleaved Lys48-linked ubiquitin chains from conjugates substrates and functioned as the deISGylating enzyme to release the mono-ISG15 from the ISGylated conjugates cellular proteins (Figure S5). PDCoV PLpro processed higher molecular weight (HMW) ubiquitin conjugates stabilized by tumour necrosis α (TNF-α)/MG132 treatment; regrettably, no significant accumulation of di-Ub products was observed (Figure S6).

### Both Macro and Ubl2 modulate the stability of the PLpro domain

**Figure 5.**
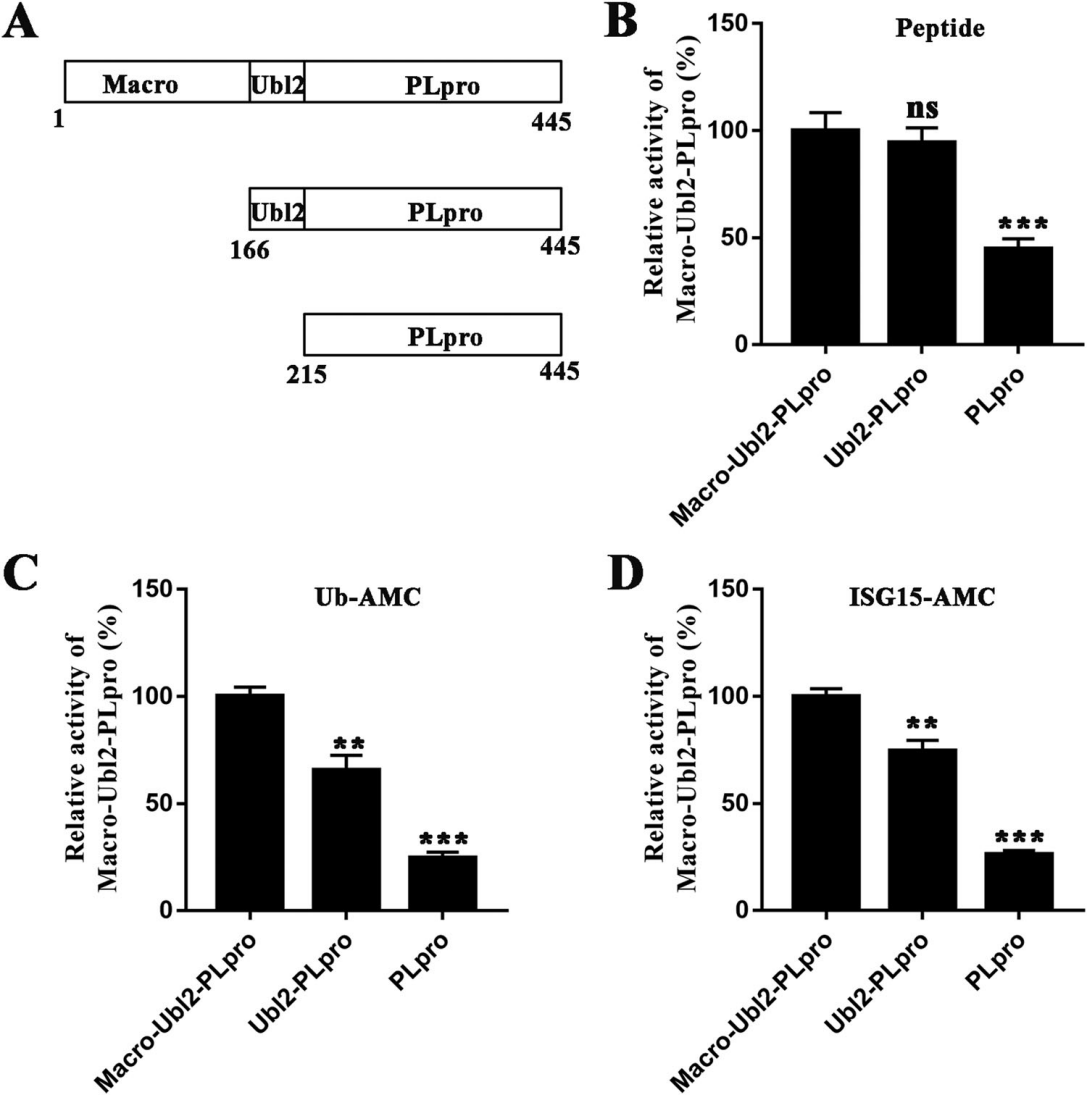
*In vitro* activity assays of PDCoV Macro-Ubl2-PLpro and truncated protein. (A) PDCoV Macro-Ubl2-PLpro and truncated constructs. (B) The hydrolytic activity assay was performed with 2 µM enzyme and 40 µM FRET peptides. The Macro-Ubl2-PLpro fluorescence intensity value was set to 100%. (C) The deubiquitinating activity assay was determined with 1 µM enzyme and 0.4 µM Ub-AMC. (D) The deISGylating activity assay was determined with 1 µM enzyme and 0.4 µM ISG15-AMC. Experiments were performed in triplicate, the wild-type fluorescence intensity value was set to 100%. The error bars represent the standard deviations for a minimum of triplicate samples. Asterisks indicate statistical signiﬁcance calculated using unpaired two-tailed student’s *t*-test, and values of 0.05 were considered statistically significant. *p < 0.05; **p < 0.01; ***p < 0.001.

The truncated constructs Ubl2-PLpro and PLpro were obtained to determine whether the Macro and Ubl2 domains alter the enzyme activity of PDCoV PLpro. A schematic domain presentation of these truncated constructs is shown in Figure 5(A). The hydrolytic activity of PLpro was reduced by approximately 50% in the absence of Ubl2 (Figure 5(B)), and the deubiquitinating and deISGylating activities of PLpro was reduced by approximately 20%, compared with of the activity of Macro-Ubl2-PLpro (Figure 5(C,D)). Similarly, deubiquitinating and deISGylating activities of Ubl2-PLpro were significantly lower than that of Macro-Ubl2-PLpro (Figure 5(C,D)). These results indicate that Ubl2 plays important role in maintaining the enzyme activity of PLpro, meanwhile Macro might participate in regulating the interaction between PLpro and host proteins.

### Unique catalytic centre of PDCoV PLpro

**Figure 6.**
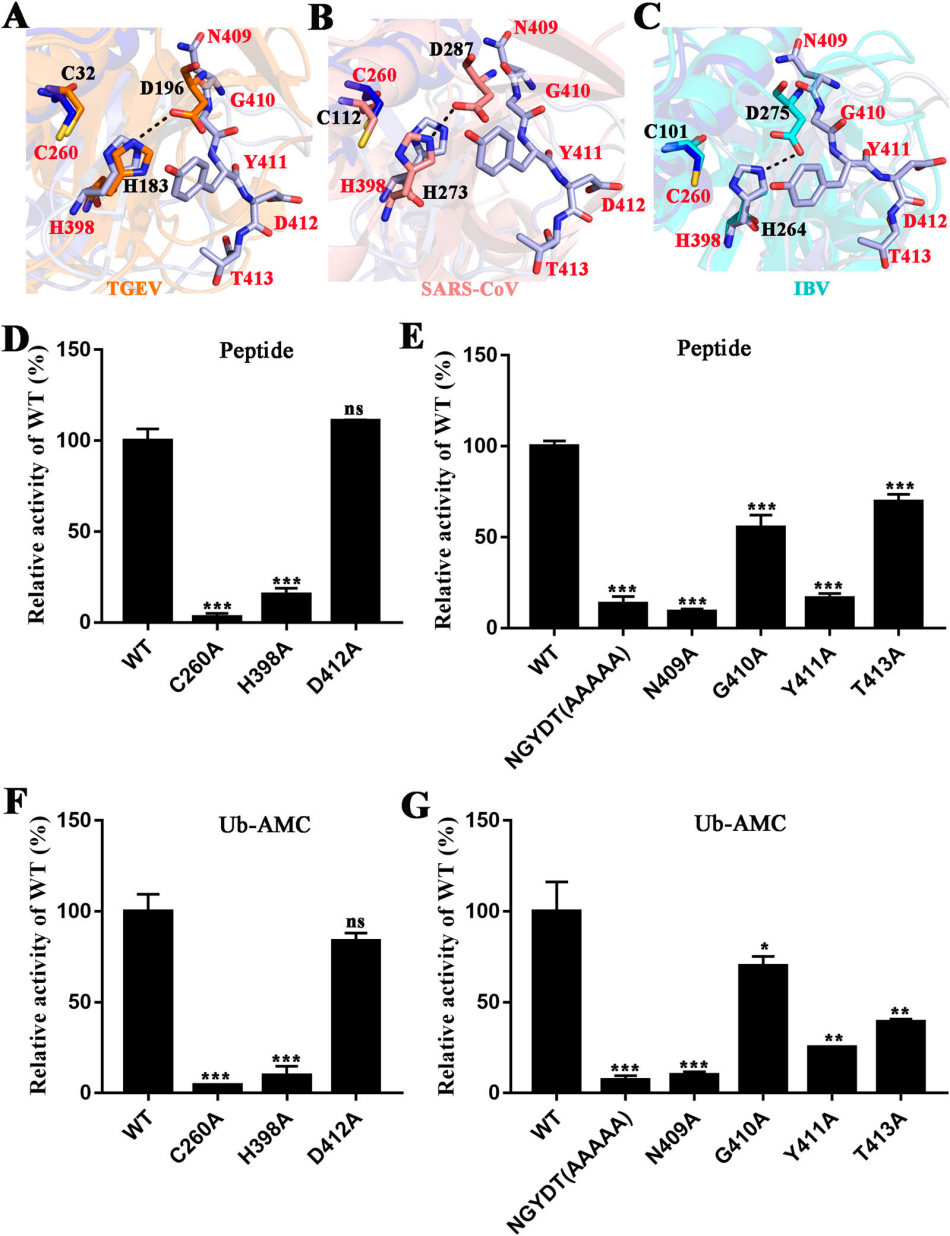
Active sites in PDCoV PLpro. (A) Superposition of catalytic residues of PDCoV PLpro (blue and light blue) and TGEV PLP1 (PDB ID: 3MP2, orange sticks). (B) Superposition of catalytic residues of PDCoV PLpro (blue and light blue) and SARS-CoV PLpro from β-CoV (PDB ID: 2FE8, salmon sticks). (C) Superposition of catalytic residues of PDCoV PLpro (blue and light blue sticks) and IBV PLpro (PDB ID: 4X2Z, cyan sticks). From A to C, C260 and H398 residues are the catalytic residues of PDCoV PLpro; N409, G410, Y411, D412 and T413 residues are the composition of the key motif. The distance between His and Asp in TGEV PLP1, SARS-CoV PLpro and IBV PLpro is indicated with black dashed lines. (D) and (E) The relative enzyme activities of PDCoV Macro-Ubl2-PLpro (WT) and mutants. The hydrolytic activity assays were performed with 2 µM enzyme and 40 µM FRET peptides as described in the Materials and Methods. The wild-type fluorescence intensity value was set to 100%. (F) and (G) The deubiquitinating activity assay was determined with 1 µM enzyme and 0.4 µM Ub-AMC. Experiments were performed in triplicate, the wild-type fluorescence intensity value was set to 100%. The error bars represent the standard deviations for a minimum of triplicate samples. Asterisks indicate statistical signiﬁcance calculated by unpaired two-tailed student’s *t*-test, values of 0.05 were considered statistically significant. *p < 0.05; **p < 0.01; ***p < 0.001.

PLPs from α, β and γ-CoV comprise the typical Cys–His–Asp triad, and the catalytic triad Cys–His–Asp of these PLPs is preformed, while the catalytic residues of the homologous structure USP7 become well aligned after binding to ubiquitin [50]. We determined that the active sites of PDCoV PLpro consist of Cys and His residues, similar to the active sites observed in many PLPs (Figure 6 and Figure S4). Cys260 is located at the base of α9 of the thumb 3.6 Å from His398, which is situated at the base of the palm domain, while the third hypothetically conserved site aspartic acid (Asp412) is not well aligned with Cys260 and His298, the position of PDCoV PLpro Asp412 is quite inconsistent among other PLPs, and the possible explanation is that the Asp412 is located on the flexible loop connecting β19 and β20 (Figure 3(A) and Figure 6(A–C)). To determine whether PDCoV PLpro was the typical catalytic triad, and whether Asp412 was the third active site, the active site mutants C260A and H398A and the third hypothetical active site mutant D412A were generated. The C260A and H398A mutants were completely inactive with the peptide substrate (Figure 6(D)). Unexpectedly, the putative catalytic triad mutant D412Ahad no effect on the hydrolytic activity (Figure 6(D)), and the results of the deubiquitinating and deISGylating activities *in vitro* were consistent with the hydrolytic activity (Figure 6(E) and Figure S7A). These results indicate that PDCoV PLpro may possess a catalytic dyad or a different third catalytic site.

The superposition of the active site of PDCoV PLpro with the active sites of the TGEV, SARS-CoV and IBV PLPs revealed that the NGYDT motif (409-413 aa) on the loop is close to Cys260 and His398 (Figure 6(A–C)). Interestingly, the position of Asn409 is approximately consistent with the third active site of other CoV PLPs (Figure 6(A–C)), in contrast, Asp412 is far from that position, which explains why the D412A mutant had no effect on the enzyme activity of the PDCoV PLpro (Figure 6(A–C)). Apart from Asn409, the benzene ring of Tyr411 inserts into the catalytic centre, due to the misaligned active sites of the PDCoV PLpro catalytic core domain, the catalytic form of PDCoV PLpro is uncertain. We tried to stabilize the loop by soaking the ligands for co-crystallization to explore the catalytic form of PDCoV PLpro; unfortunately, we failed to obtain the crystal complex. Then, we mutated the NGYDT motif to AAAAA to explore the third catalytic site (Figure S4). To our surprise, the mutant almost completely lost its hydrolytic activity and deubiquitinating activity (Figure 6(F,G)), consistent with the effects of the C260A and H398A mutations. This result reveals that the NGYDT motif is necessary for enzyme activity. The single N409A, G410A, Y411A and T413A mutants were obtained to determine the key residue of the motif that regulates hydrolysis. Both the hydrolytic activity and deubiquitinating activity of N409A are similar to those of C260A and H398A mutants, while the T413A mutant shows slightly decreased hydrolytic activity and deubiquitinating activity compared with the wild-type protein (Figure 6(F,G)), as the same as the results of the deISGylating activity *in vitro* (Figure S7). Based on these results, the catalytic core of PDCoV PLpro is unique among the reported CoVs PLPs, the homologous proteins EAV PLP2 [35] and Ubp6 (in yeast) also adopt the Cys–His–Asn catalytic triad [48].

## Discussion

### Schematic of the PDCoV Macro-Ubl2-PLpro structure

Our study revealed that the multifunctional domain crystal structure of PDCoV nsp3 from the δ-genus contained both Macro and Ubl2-PLpro, is similar to a previous report [10]. In contrast, SARS-CoV nsp3 contains the Mac II, Mac III and DPUP domains [51], MERS-CoV nsp3 contains Mac II and DPUP [10], and MHV contains DPUP [25]. Meanwhile, only one PLpro is present in SARS-CoV, MERS-CoV, IBV and PDCoV, corresponding to the β-, γ-, and δ-genera, respectively, and two PLPs are present in TGEV and MHV from the α- and β-genera, respectively [5,10]. CoVs from different genera have different nsp3 domain organizations, indicating that CoV nsp3 from different genera may have evolved differently. Nsp3 of PDCoV is the smallest among the CoVs. These results also suggest that CoVs can survive with the most concise and effective functional domains, such as PDCoV nsp3.

Phylogenetic relationships were examined to explore the evolution of nsp3 in coronaviruses. Evolutionarily, the first branch shows a close relationship between α and β genera that is similar to the relationship between the γ and δ genera in the second branch (Figure S8). Researchers have not determined the origins of the first CoVs; however, 3CLpro, helicase, RNA-dependent RNA polymerase (RdRp) and nucleocapsid protein (N) show the same phylogenetic tree branches and order as nsp3 [52]. No obvious pattern of evolution was observed for the macrodomain and PLpro, probably due to their different hosts. Thus, for the survival of the fittest in the host, CoVs may have evolved different functional domains in nsp3. The macrodomain widely exists in various organisms, and PLpro mimics deubiquitinase in other organisms, which provides evidence in support of this theory [12]. ORF1a and ORF1b of PDCoV encode two polyproteins pp1a and pp1b, which are proteolytically cleaved to 15 nonstructural proteins by nsp3 and nsp5 [52,53]. Due to the lack of nsp1 in PDCoV, the putative protease cleavage sites of PDCoV nsp2/3 and nsp3/4 are all AG for PDCoV, WECoV HKU16, SpCoV HKU17, MRCoV HKU18, and CMCoV H21, while the putative protease cleave sites are only relatively highly conserved among these delta-coronaviruses [52]. Compared with nsp3 from other CoVs, PDCoV nsp3 is relatively small, and our structure also reveals that the PDCoV PLpro is obviously smaller than that of other CoVs, except for the Macro, which belongs to the conserved replicase domains for species demarcation; the whole PDCoV nsp3 has a low homology to other CoVs [52].

The PDCoV Macro adopts a typical α/β/γ sandwich fold, as observed in the other three coronaviral genera that belong to the macroH2A family. High affinity with ADP-ribose or poly-ADP-ribose is a well-known characteristic of the conserved CoVs Macros [17,21,22]. The typical solvent-exposed cleft on the surface of PDCoV Macro and the moderately conserved putative active site indicate that the Macro of PDCoV may have the same enzyme activity. Unfortunately, attempts at soaking and cocrystallization failed to yield crystals of the complexes.

Currently, except for the reported MHV tandem structure [25], no reported structures contain both Ubl2 and the functional domain before Ubl2. The superposition of our whole structure with SARS-CoV Ubl2 and MHV DPUP-Ubl2-PLpro revealed the offset Ubl2 of PDCoV, which is a unique structural feature among CoVs. Ubl2 is necessary to stabilize the structure of PLpro of MHV [37], and the flexibility of Ubl2 may regulate the innate immune response [53]; thus, the unique orientation of PDCoV Ubl2 plays an important role in antagonizing the host innate immune response.

Due to the short fingers of PDCoV, the whole PLpro structure is much smaller than that of SARS-CoV and IBV, and the structural differences may indicate functional differences. Several differences in the palm and finger domains have been observed among the PLPs (Figure 3), and these differences lead to the more compact and non-stretchable structure of PDCoV PLpro, which may affect its binding to substrates.

### Enzyme activity of PDCoV PLpro

DUBs play an important role in modulating the innate immune response. CoV PLPs function as DUB enzymes by downregulating interferon (IFN) secretion and interferon-stimulated genes (ISGs) in the anti-antiviral response of host cells. For example, SARS-CoV PLpro and NL63 PLpro have deISGylating activity, and a like ubiquitinated protein is associated with the innate immune response [25,33,54]. Here, we report for the first time that PDCoV PLpro possesses hydrolytic, deubiquitinating and deISGylating activities, and the enzyme activity of PDCoV PLpro is significantly lower than SARS-CoV [49] and TGEV PLPs [26], although the deubiquitinating activity of PDCoV PLpro is similar to USP7 [50]. The structural and functional features of PDCoV PLpro reveal that the PLpro of PDCoV may use similar mechanisms of protection against the antiviral response of the host.

Nsp3 is a large nonstructural protein with multifunctional domains whose regions should all mediate a coordinated effects; however, current studies on nsp3 mainly focus on specific regions, and the mechanism of the collaboration and mutual assistance of the individual domains remains unclear [5]. Comparison of the hydrolytic activity and deubiquitinating activity between Macro-Ubl2-PLpro and truncated Ubl2-PLpro and PLpro revealed that both Macro and Ubl2 are important for stabilizing the enzyme activity of PLpro and indicated that the entire structure of nsp3 may function better. An interaction interface is observed between Macro and PLpro in the crystal structure, whereas Mac-Ubl2-PLpro is a monomer in solution; the amino acids that participate in the interaction are also not conserved in CoVs (Figures S3 and S4). Therefore, the interaction forces between Macro and PLpro in PDCoV may be very weak. According to a recent report, six copies nsp3 is the component of the DMV molecular pore with the N-terminal Ubl1 residing in the prongs and a channel existing in the sixfold axis, which may be associated with the release of viral genome RNA into the cytoplasm [55]. The Ubl1 of nsp3 binds ssRNA and interacts with the N protein [5], while the conformation of SARS-CoV Ubl1 changes in the presence of RNA [56]. Several reports indicate that the morphological conformation of DMVs may change dynamically [5,10], and nsp3 may serve as a driven multi-functional linkage switch component in a manner that PLpro may briefly interact with Macro to pull the Ubl1 to transport RNA into the cytoplasm. Correspondingly, nsp3 exert different function by switching the form in this dynamic conformation.

With the outbreak of the novel coronavirus in 2019, effective vaccines and drugs for prevention and treatment are hot research topics. A series of reports examining the SARS-CoV-2 indicated that nsp3 is a potential drug target [57–59], although the exact mechanisms of the functions of each region of nsp3 remain unclear, and a more multifunctional domain structure will be required. Hence, the tandemly linked domains of PDCoV nsp3 may provide insights for the further study of SARS-CoV nsp3.

### The uniqueness of the catalytic core of PDCoV PLpro

All the enzyme activity core of the reported PLPs of CoVs adopt the typical Cys–His–Asp triads [24–27], whereas in the Arteritis viruses, the catalytic site of EAV PLP2 comprises the Cys–His–Asn triad [35], and the third active site of Ubp6 in yeast is replaced by Asn [48]. In our study, we determined two conserved active sites of PLpro, Cys260 and His398; these sites are consistent with the reported enzyme active sites of CoV PLPs [24–27]. However, the third putative and conserved Asp412 is not the active site, and the hydrolytic activity, deubiquitinating and deISGylating activities of the D412A mutant are similar to those of the Macro-Ubl2-PLpro. Unexpectedly, Asn409 occupies the position that is consistent with the third active site Asp of other CoV PLPs, and replacing this Asn with Ala clearly decreases the enzyme activity of PDCoV PLPro. Similarly, the Y411 exerts a significant effect on stabilizing the structure of PDCoV PLpro, because imidazole ring of His398 and the benzene ring of Y411 form the π-π conjugate bond, located in the catalytic centre. These results reveal that PDCoV PLpro may adopt a unique catalytic mode. Then, we tried to stabilize the loop NGYDT 409-413; unfortunately, we failed to obtain the crystal complex of PDCoV PLpro with human ubiquitin, perhaps due to the interaction between Macro and PLpro. The differences in the catalytic core of PLpro may lead to functional differences, and the unique catalytic core that may influence substrate binding with PDCoV PLpro requires further exploration.

## Supplementary Material

Supplementary_materials-1.docx

## Data Availability

The coordinates and structural characteristics of PDCoV Macro-Ubl2-PLpro were submitted to the Research Collaboratory for Structural Bioinformatics (RCSB) under PDB accession number 6LNO.
