# Supplementary material for: Structure of the multiple functional domains from coronavirus nonstructural protein 3
Source: Emerg Microbes Infect. 2021 Jan 17;10(1):66–80. doi: 10.1080/22221751.2020.1865840 (PMC7832007; doi:10.1080/22221751.2020.1865840)
Supplement: Supplementary_materials-1.docx [file TEMI_A_1865840_SM0046.docx]

**Supplementary materials**

**
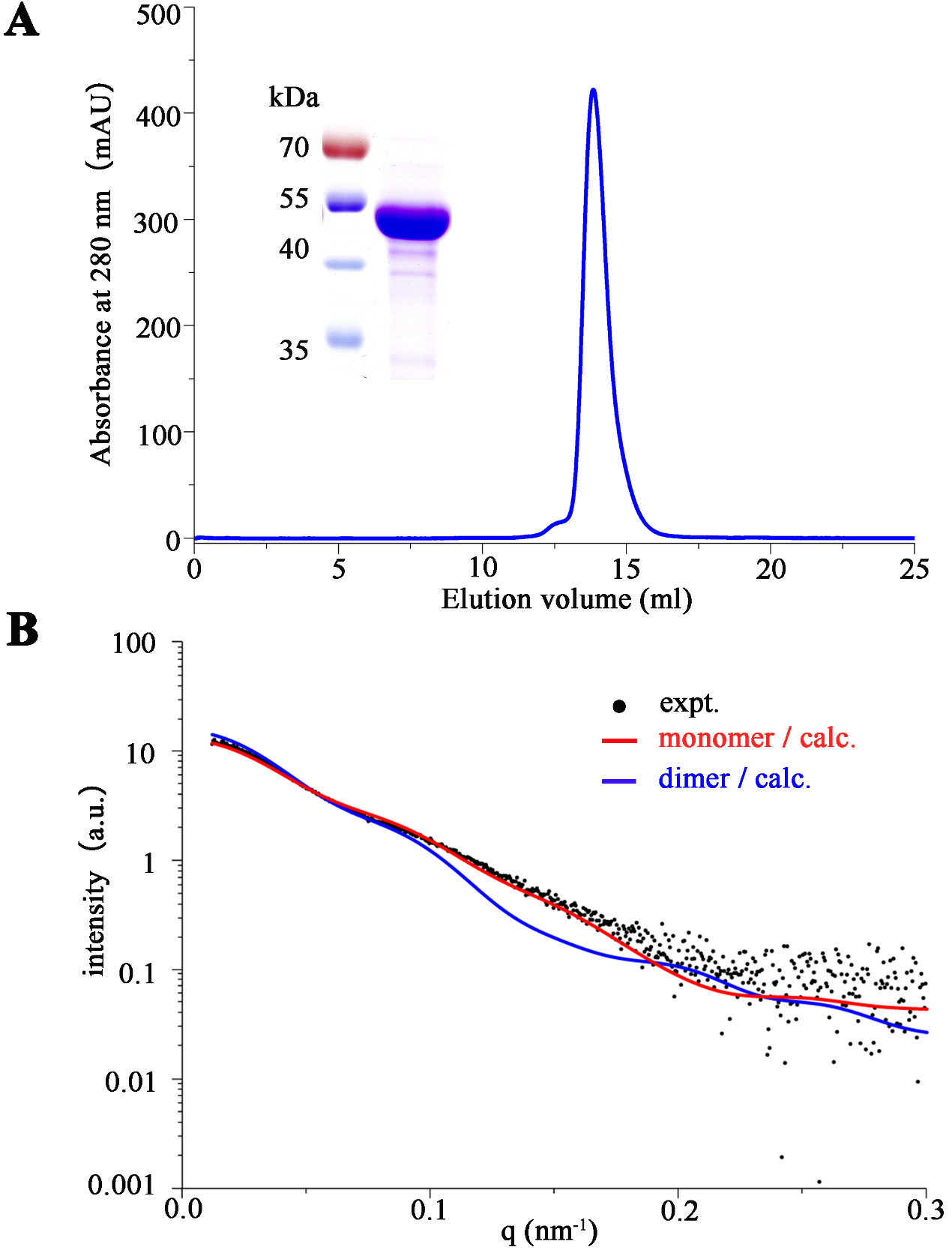
**

**Fig S1. PDCoV Macro-Ubl2-PLpro is a monomer in solution.** (A) Analytical gel filtration of PDCoV Macro-Ubl2-PLpro protein. The 280 nm absorbance curve from the Superdex 200 10/300 Gel column and the SDS-PAGE migration profile of the pooled sample are shown. (B) Small-angle X-ray scattering analysis. Comparison between experimental and predicted SAXS curves on a semiology scale in the low-frequency region up to 15 Å.


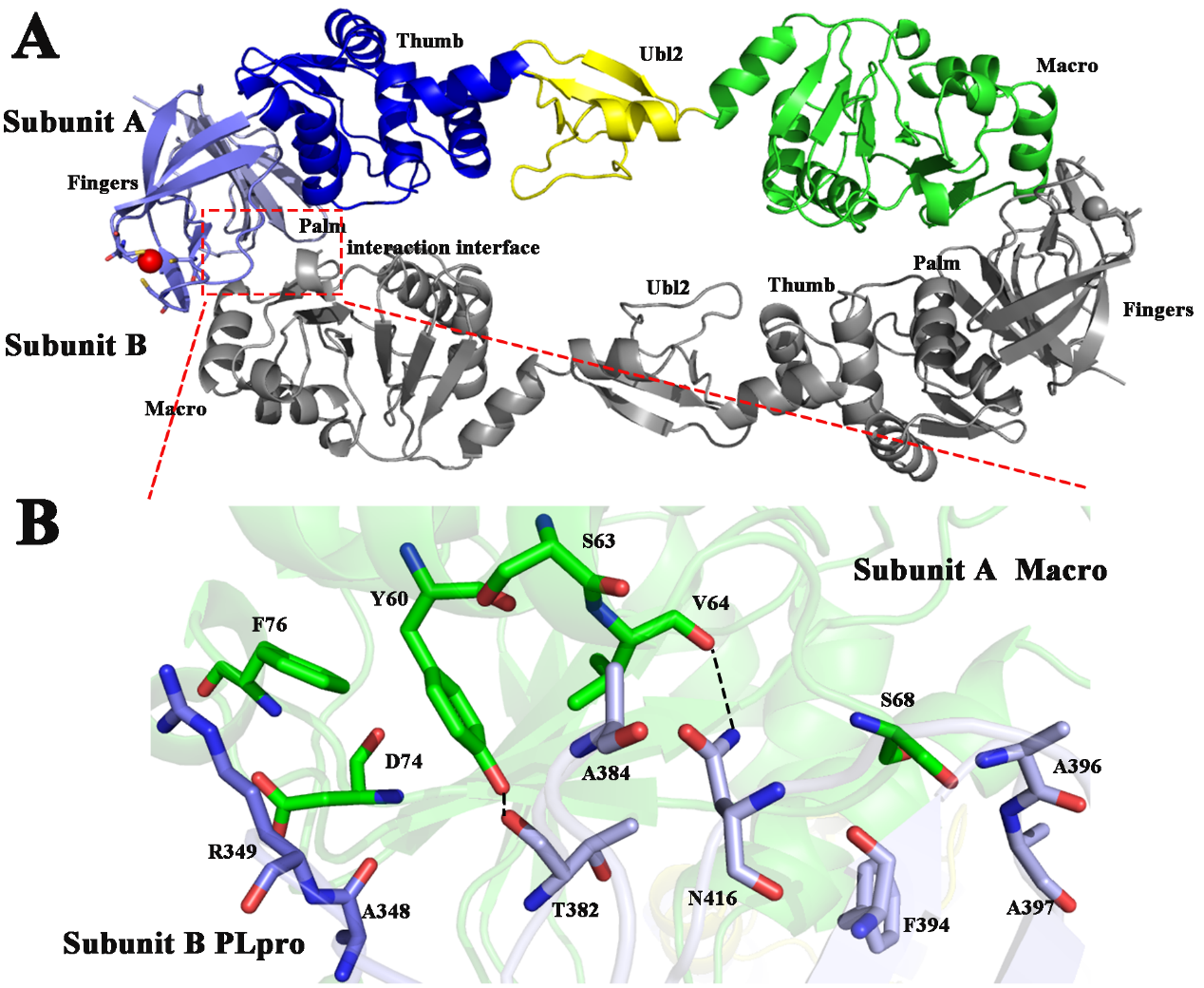


**Fig S2. PDCoV Macro-Ubl2-PLpro forms a dimer in the asymmetric unit.** (A) Crystal homodimer of PDCoV Macro-Ubl2-PLpro. Subunit A is shown in multiple colors, and Subunit B is shown in gray. The Macro domain is shown in green, the Ubl2 domain is shown in yellow, the thumb domain is shown in blue, the finger domain is shown in slate, and the palm domain is shown in light blue. The interaction interface is highlighted with a red dashed line. (B) Stereo view of the interaction interface between the Mac and PLpro domains; the color coding is the same as in A. The hydrogen bonds are shown as black dashed lines.


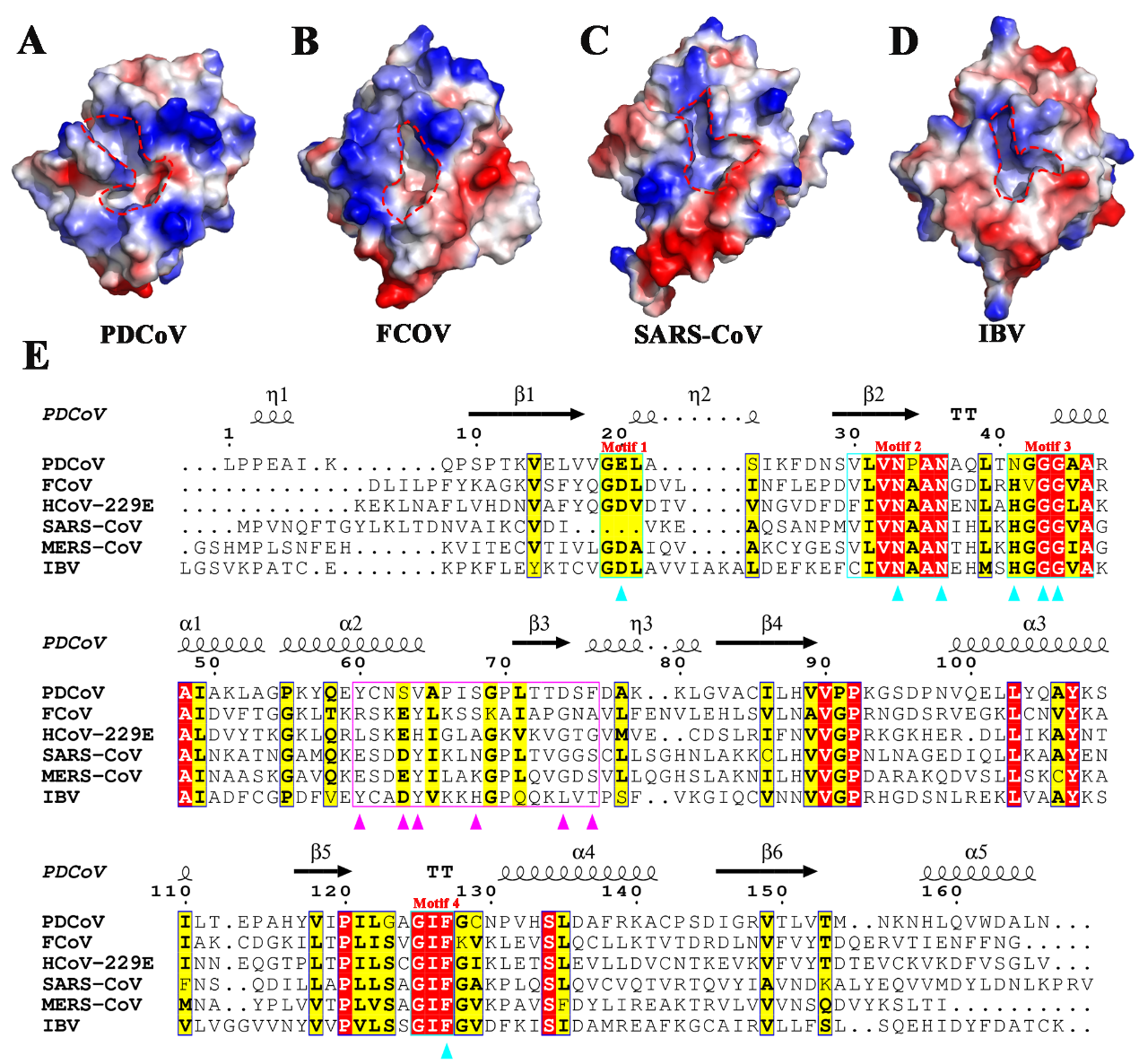


**Fig S3. Surface distribution of electrostatic potential and sequence alignment of Macro among four CoV genera.** (A)-(D) Macros of PDCoV, FCoV, SARS-CoV and IBV are shown as a molecular surface model colored according to electrostatic potential (red for negatively charged regions and blue for positively charged regions). The potential binding pocket of ADP-ribose is highlighted with a red dashed line. The PBD IDs of FCoV, SARS-CoV and IBV Macros are 3ETI, 2ACF and 3EWP, respectively. (E) Sequence alignment of coronaviral macrodomains. α-CoV, FCoV and HCoV-229E; β-CoV SARS-CoV and MERS-CoV; γ-CoV IBV; and δ-CoV PDCoV. The following sequences from NCBI were used to create the sequence alignment (abbreviations and NCBI accession number): PDCoV KP757891.1; FCoV, Q98VG9; HCoV-229E, P0C6U2; SARS-CoV, P0C6X7; MERS-CoV, K9N638; and IBV, P0C6V5.1. Identical residues are highlighted in red, and conserved residues are shown in yellow. These sequences were aligned using ClustalW2, and the alignment was drawn with ESPript3.0. The key residues for potential binding to the ADP-ribose activity sites and dimerization sites of PDCoV Macro are marked with cyan and purple triangles at the bottom, respectively. The four conserved stretches of residues are highlighted in cyan blocks. Secondary structural elements of the PDCoV Macro are marked on the top of the alignment (helices with squiggles, β-strands with arrows, and turns with TT letters).


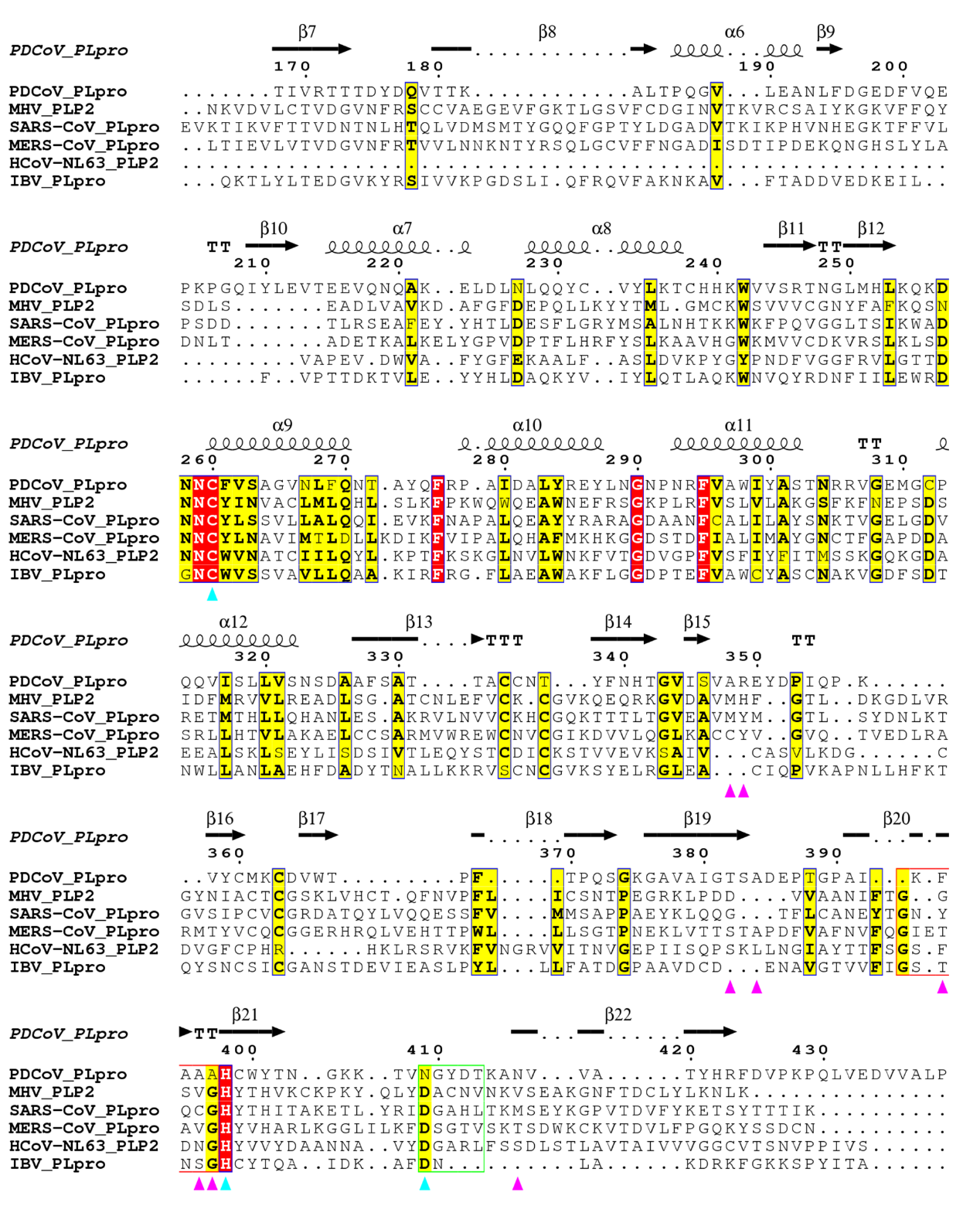
**Fig S4. Sequence alignment of PLPs among coronaviruses.** α-CoV, MHV; β-CoV SARS-CoV and MERS-CoV, HCoV-NL63; γ-CoV IBV; and δ-CoV PDCoV. The following sequences from NCBI were used to create the sequence alignment (abbreviations and accession numbers): PDCoV, KP757891.1; MHV, NP_045298; SARS-CoV, ADC98008.1; MERS-CoV, YP_009047215.1; HCoV-NL63, AFD98832.1 and IBV, P0C6Y1. Identical residues are highlighted in red, and conserved residues are shown in yellow. The sequences were aligned using ClustalW2, and the alignment was drawn with ESPript3.0. The key residues are marked with triangles at the bottom: enzyme activity sites, cyan; PDCoV PLpro dimerization sites, purple; and the NGYDT motif, green. The block loop 2 and motif are framed with red and green lines, respectively. Secondary structural elements of PDCoV Macro are marked on the top of the alignment (helices with squiggles, β-strands with arrows, and turns with TT letters).


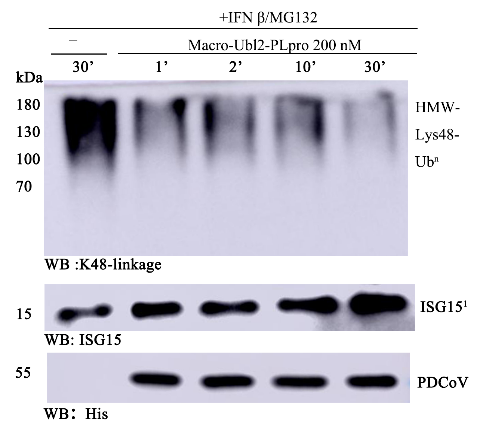


**Fig S5. DUB and deISGylating activities in lysates.** HeLa cells were treated with human IFN β and MG132, and cleavage of HMW-Ub-Lys48 and ISG15-conjugates in lysates incubated with 200 nM PDCoV Macro-Ubl2-PLpro were analyzed.


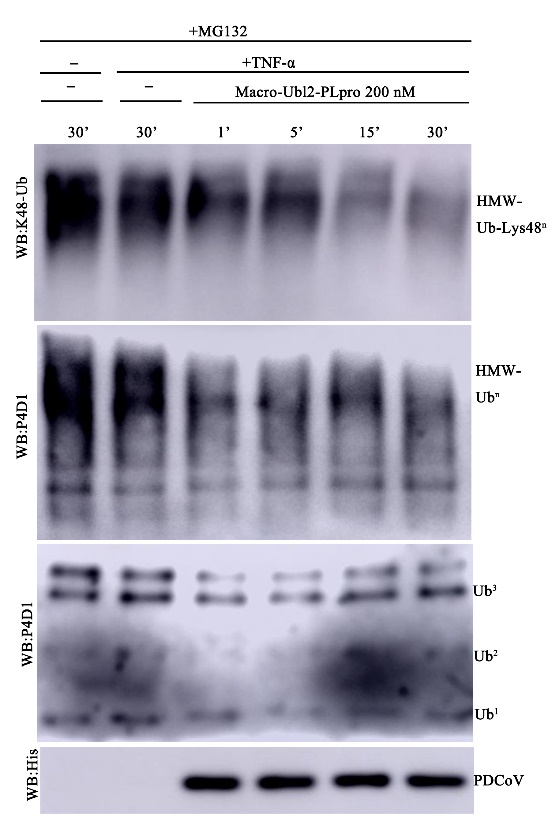


**Fig S6. Ub-conjugate cleavage in lysates.** HeLa cells were treated with TNF-α and MG132. HMW-Ub-Lys48 and Ubiquitin-conjugates in lysates incubated with 200 nM PDCoV Macro-Ubl2-PLpro were analyzed.


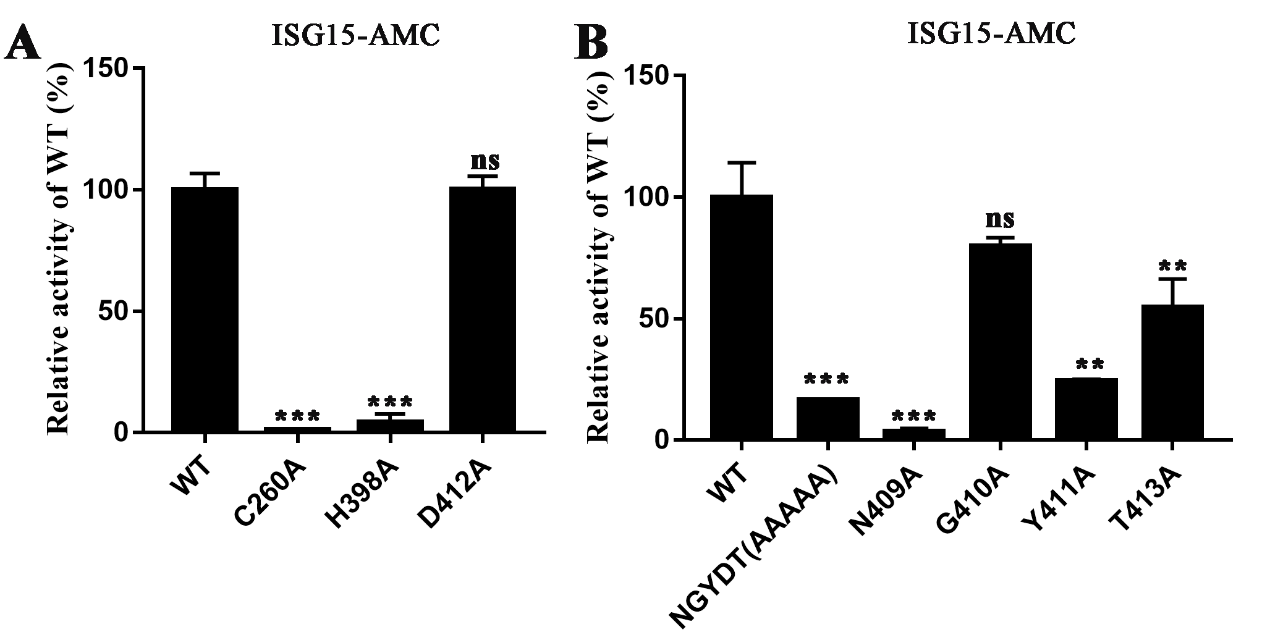


**Fig S7. PDCoV Macro-Ubl2-PLpro(wild-type) and mutants in vitro deISGylating activity assays.** The assays were determined with 1 µM enzyme and 0.4 µM ISG15-AMC. Experiments were performed in triplicate, the wild-type fluorescence intensity value was set to 100%. The error bars represent the standard deviations for a minimum of triplicate samples. Asterisks indicate statistical signiﬁcance calculated using unpaired two-tailed Student’s t test, and values of 0.05 were considered statistically significant. *, P < 0.05; **, P < 0.01; ***, P < 0.001.


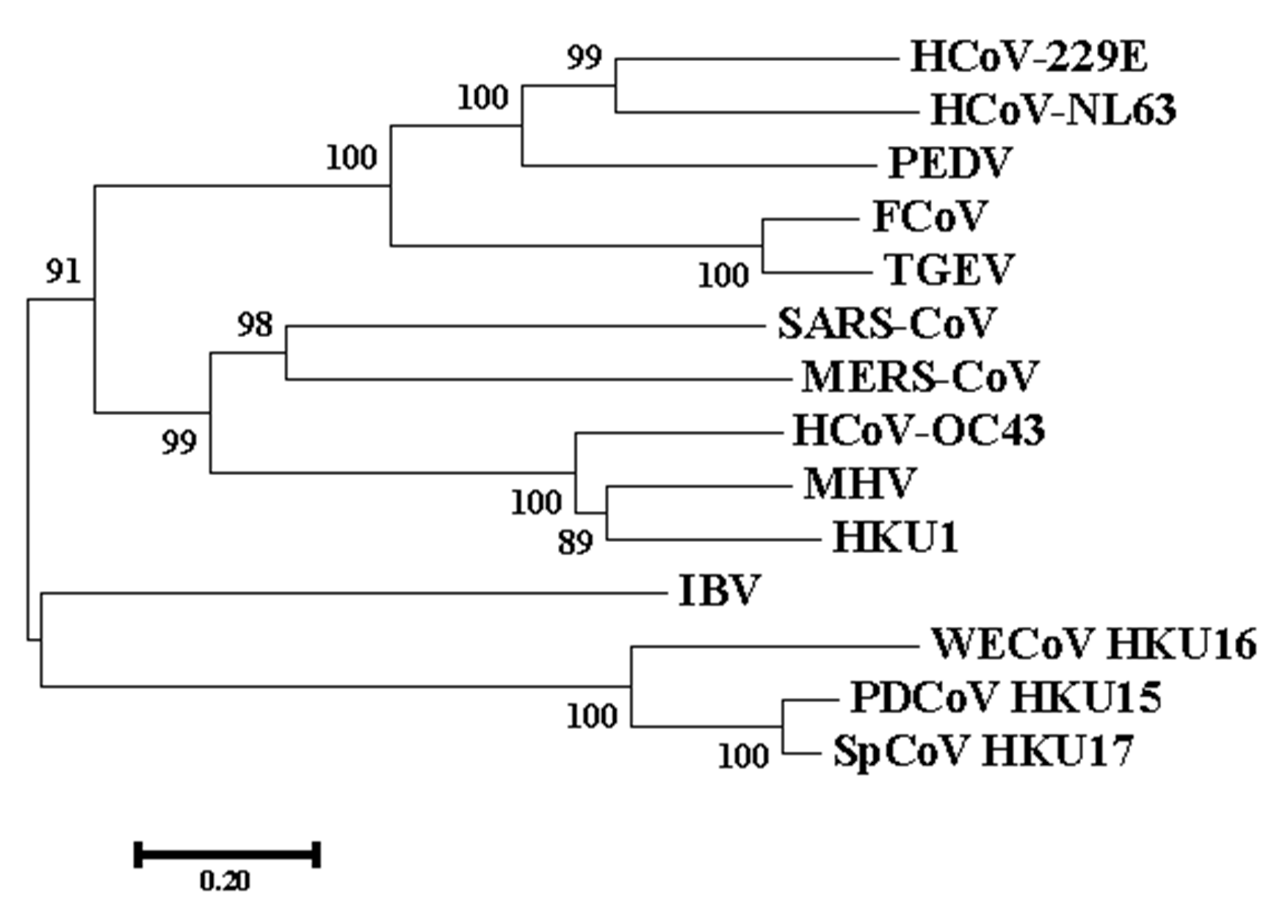


**Fig S8. The phylogenetic tree was analyzed using the distance-based neighbor-joining method in the MEGA package.** The different sub genotypes are indicated.
